# Academy of Stomatology

**Published:** 1900-08

**Authors:** 

**Affiliations:** Academy of Stomatology


### ACADEMY OF STOMATOLOGY.

AT the regular meeting of the Academy of Stomatology, held March 27, 1900, the President introduced the essayist for the evening, Dr. B. Holly Smith, of Baltimore, Md., who read his paper entitled "Fetor from a Dental Stand-Point."

(For Dr. Smith's paper, see page 514.)

*The President.*—Gentlemen, I am sure that you will agree that we have all listened to an extremely interesting and able paper on a very important subject. It is a subject to which, perhaps, we do not give the attention that we should as a profession. I hope that we shall have a general discussion of this matter. I am sure you are all interested in it, and all must have had some experience which will be of benefit to us here.

*Dr. Brubaker.*—It seems to me that all the statements that have been made have been based on well-established physiological and pathological facts.

Independent of the local condition of the mouth which has been alluded to, there can be no doubt that much of the disagreeable fetor of the breath is dependent upon the putrefaction of proteid material which occurs in the small intestine. Owing probably to some indiscretions in diet, there is gradually established in the small intestine, more particularly in the neighborhood of the biliary passages, a catarrhal condition which interferes with the proper elimination of bile and probably of pancreatic juice, and, in consequence of this, the proteids undergo various stages of putrefaction, with the development of compounds which are extremely fetid, such as skatol, indol, etc. Some of these compounds, after being absorbed from the intestine, are eliminated under various forms, but there is not much doubt that some of these putrefactive products are absorbed into the blood and eliminated by the pulmonary mucous membrane, and, in this way give a very decided odor to the breath. These decompositions which take place in the small intestines are very readily recognizable by well-known clinical tests, and can, to a very great extent, be obviated by treatment of this part of the intestinal canal.

In this connection I think it may not be out of place to make a suggestion with reference to the treatment. The treatment of the intestine is very simple, and consists essentially in the removal of this catarrhal state, which is accomplished to a very great extent by the use of pulverized sodium phosphate, given in half-teaspoonful doses with plenty of water daily, especially in the morning. It has an admirable effect, stimulating the intestinal tract and biliary passages. A pill administered at night as required, and consisting of one grain of blue mass, one grain of aloe, and one-quarter of a grain of podophyllin, is one of the best combinations for the removal of those putrefactive changes in the small intestines upon which fetor of the breath very frequently depends. The drinking of an additional quantity of water assists elimination of these odorous emanations. I think that every dentist is privileged to prescribe remedies as simple as those I have mentioned, and I am quite sure that if they are applied this condition of the breath will be largely removed.

*Dr. Schamberg.*—I applaud the sentiments that have already been expressed in this valuable paper. There are one or two points that come to my mind to which I would like to call the attention of the society,—namely, the excessive fetor that is produced by the use of tannic acid in the mouth for the checking of hemorrhage. Only recently I had occasion to assist Professor Cryer, at the University, in an operation upon a necrosed maxillary bone, in which there was a considerable amount of hemorrhage and tannic acid was used as the hæmostatic. In this instance we noted a most horrible fetor. It was simply unbearable upon changing the dressing, and was sufficient to make the patient feel temporarily sick. I was quite sure she had been inhaling these obnoxious odors up to the time of the changing of the dressing. I must say that in other particulars the paper is highly important. I myself experience after eating a meal a most horrible odor if I nap after that meal, and many thoughts have suggested themselves to me as to the cure of such a condition. One is the use of some form of lozenge which one may take as a confection and in which would be incorporated certain drugs which have antiseptic properties that would act to overcome the fetor of the breath, and, at the same time, neutralize the acidity of the mouth. Of course, this at first seems rather contrary to ideas of the dental profession regarding the effect of confections upon the teeth. At the same time, I imagine that such a lozenge could be made palatable and even inviting to even young children, and I imagine that much dental caries could be checked in that way.

*Dr. A. H. Thompson.*—I have been very much interested in the paper and have benefited by it, as we all have been, and I should like Dr. Smith to have gone very much more into detail with regard to such antiseptic care of the mouth as would be desirable for us to advise our patients to associate with ordinary hygiene.

We know that accumulations about the teeth give rise to offensive odors, even with persons in the best of health, and that constant care is required to remove particles that may decompose. I think we do not yet have the proper method of what we might call the sanitation of the mouth, nor that we prescribe for our patients what is really ideal hygienic care. Of course, we have some antiseptic washes that can be used, but something more is needed.

*Dr. Pierce.*—I want to thank Dr. Smith for the paper, and say that I believe with Dr. Thompson that we have quite a field for the introduction of good antiseptic washes. I have been impressed for some time with the advantage that may be obtained by their proper use, and hope for the production of some wash or washes that may be advantageous in fetor due to local conditions.

*Dr. Gaskill.*—I was very much interested in the paper, and think attention should be especially directed to the offensiveness of badly constructed bridges and crowns, particularly the so-called "saddle-bridge." Food and foreign substances will crowd beneath it. If we had a perfectly hard surface to deal with it might be different, but the mucous membrane yields and the saddle does not fit. In one case a saddle-bridge had been in the mouth for one year. The patient had paid six hundred dollars for it. The saddle was carried back from the cuspid for a distance the width of three teeth on one side, and there was a strip of gold crossing the palate, with a piece of bridge-work on the other. This made a shelf for the deposit of food. When that piece of work was shown to the patient she said, "You could not pay me a thousand dollars to have that put back into my mouth." That was done by a man of wide reputation, and I think he should have known better.

*Dr. Jameson.*—I have enjoyed Dr. Smith's paper very much, and believe he has done good service in calling our attention to these conditions, which should meet with more thorough and general recognition and systematic treatment at our hands.

I have noticed a number of times the fetor arising from diseases of the antrum. When the antral troubles are removed, of course the fetor disappears.

*Dr. William Trueman.*—I have listened with a great deal of attention to the essayist, and was particularly impressed by the suggestions which he made and the way in which he broadens the field of dental science. It is not sufficient that we understand the parts upon which we work, but we have opportunities constantly offered us to be of very great assistance not only to the patient, but to the patient's physician. The doctor has related cases that had been under the care of specialists who had utterly failed to discover the causes of the trouble. The common idea is to mask fetor of the breath, and you all recognize that as dangerous. The next idea is to combat it by some agent that shall neutralize the agent that is producing the bad odor. The doctor suggests this not to be the proper way, but to find and remove the cause, restore the parts to a healthy condition, and then the odor at once ceases.

*Dr. Cupid.*—This is such a practical question that it interests us all. If we do not understand particularly the cause of the trouble we certainly cannot work out its removal or overcome the condition. I think, as has been said by the essayist and other writers previously, that there should be attempted the mechanical removal of this deleterious substance—no doubt waste material—which has been retained in the tissues, particularly in the furred tongue. The dead epithelial layers which should be constantly thrown off are being retained, chemical decomposition is going on, leading up to a profound putrefaction, which necessarily produces intense and persistent odors, and if we can remove them mechanically it is a great step towards overcoming the trouble. If we can go back and remove the cause of the trouble it is still better.

I wish to speak of two different systems that I have followed very carefully and partly successfully. These are the medicinal treatment with permanganate of potassium and the mechanical with the tooth-brush. I have found that thorough scraping of the furred tongue and the mucous surfaces has resulted in great improvement, not only in the case of my patients, but with myself, by the removal of collections of dead epithelial tissue and substances that have been retained about the fauces. In every case where I find fetor I invariably prescribe it, together with the use of permanganate of potassium in dilute solution.

*Dr. Gaylord.*—I feel very grateful to the essayist for the paper this evening. It has been very helpful to me and has broadened my ideas.

It seems to me that one point has been overlooked in the matter of fetor, and that is the accumulation of calculus about the teeth. It is one of the most important things that come under the eye of the dentist, and which, I fancy, is more or less neglected by the profession, partly owing to its disagreeable nature; but we cannot conscientiously dismiss a patient without thoroughly removing this substance. So long as it remains we cannot expect a clean, wholesome mouth.

*Dr. Rice.*—The subject of the removal of particles of food that have been retained in the interdental spaces has been dwelt upon, and I am surprised to find that the atomizer has not been spoken of. The mechanical removal of these particles of food is necessary for a lessening of this fetor. I have found that the use of the atomizer is very efficacious, and that the proper projection of the antiseptic upon the tissue is more important than the antiseptic.

*Dr. Kirk.*—I should like to call attention to the point that a fetor of the breath is not always indicative of a pathological condition. In all those conditions where the salivary secretion is suppressed, either temporarily or for a length of time, there is developed, as the essayist has indicated, a tendency to putrefactive changes in the mouth, and this tendency may be developed in a perfectly healthy individual during the night. I think that all who have had the experience of raising children have noticed that even in the mouth of a perfectly healthy infant, where there is no evidence of any caries or other pathological condition, if there is a tendency to mouth-breathing at night, as there may be for a short time, it is quite a common thing for a slight heaviness or disagreeably fetid breath to be developed. It will almost immediately pass away as soon as the salivary secretion is re-established. The reason for this would seem to be that the germs of putrefaction do not develop so readily in a mouth in which the saliva is rapidly or normally flowing.

There is another condition which has not been fully dwelt upon, and that is the relation of food-habit to fetor of the breath. It is undoubtedly true, as alluded to by Dr. Brubaker, that the digestion of large quantities of proteid food is more apt to produce this odor of the breath than where a vegetable diet is used. I think that may be accounted for by the fact that the activity of the salivary glands is perhaps more stimulated by a vegetable diet than a proteid one, with consequent inhibitory action upon the germs of putrefaction by saliva in quantity.

*Dr. B. Holly Smith.*—I think it has been well said that the importance of oral sanitation, and the means of carrying it out, is a thing that should be dwelt upon. I recall with some chagrin and mortification a patient who came to me some years ago with a mouth in almost perfect condition. The mucous membrane was everywhere of a beautiful, healthy tint. The teeth were white and clean, but at the angles the gum had been brushed away from the cuspids and bicuspids to some extent by what I supposed was a very stiff brush. I counselled the young woman to avoid the use of this stiff brush. She told me then that she used the brush quite a good deal on the soft parts, and her teeth were beautiful and her mouth was in a nice condition. I have never had the courage to say to that young woman what I must say to her when I have a settlement with my conscience, that I did her a great injury by warning her to be careful in the use of her brush. I suggested a softer brush, and now she comes to see me twice a year, and I have calculus to remove and her mouth is not in as healthy a condition as before. There is every evidence of neglect on her part, and I feel that I am a party to it. I now frequently instruct my patients to clean out about the palate and the little circumvallate papillæ, which often grow up like mushrooms. In the case I spoke of, where they were obliterated by the electric cautery, the woman was a charming lady, who occupied a good social position, and after I began to see something of her family and friends they talked about "the great misfortune." Really, it seemed so desperate that the young lady had such a bad breath that they did not like to talk to her about it, and it was a source of mortification and embarrassment to all. I had never read anything along this particular line, and I do not know that Dr. Thomas, who assisted me in the operation, had either, but we decided that these little valleys about the fungiform papillæ seemed to fill and retain enough fetid matter to impregnate every breath. Since then I have never had occasion to use the cautery, but I have called the attention of my patients to the use of the broad brush for cleaning the tongue. I have no doubt that the vigorous, though careful, cleaning of the tongue and mucous membrane will aid in the production of a healthy condition.

I am sure that it is more than gratifying for me to hear so many kind words of commendation from the members of this society, and it has been a very great pleasure for me to be with you.

Otto E. Inglis,
*Editor Academy of Stomatology.*

